# Network-level effects of optogenetic stimulation in a computer model of macaque primary motor cortex

**DOI:** 10.1186/1471-2202-15-S1-P107

**Published:** 2014-07-21

**Authors:** Cliff C Kerr, Daniel J O'Shea, Werapong Goo, Salvador Dura-Bernal, Joseph T Francis, Ilka Diester, Paul Kalanithi, Karl Deisseroth, Krishna V Shenoy, William W Lytton

**Affiliations:** 1 Department of Physiology and Pharmacology, SUNY Downstate Medical Center, Brooklyn, NY, USA; 2 Complex Systems Group, School of Physics, University of Sydney, Sydney, NSW, Australia; 3 Neurosciences Program, Stanford University, Stanford, CA, USA; 4 Department of Bioengineering, Stanford University, Stanford, CA, USA; 5 Ernst Strüngmann Institute, Frankfurt, Hesse, Germany; 6 Department of Neurosurgery, Stanford University, Stanford, CA, USA

## 

Optogenetics is a potent tool for performing precise perturbations to ongoing cortical dynamics in behaving primates. However, only small numbers of neurons can be recorded simultaneously. In this work, we present a biomimetic spiking network model of macaque primary motor cortex (M1) in order to explore network-level effects of optogenetic stimulation – namely, how synaptic connections modulate the optogenetic stimulus response, and how optogenetic stimulation affects interlaminar information flow. Experimental data were recorded from M1 in a male macaque. Optogenetic stimulation targeted excitatory neurons, likely preferentially affecting deeper layers, via the excitatory opsin C1V1TT, with either continuous (200 ms duration) or periodic (20, 40, or 80 Hz) pulses of green light (561 nm) [1]. The network model consisted of 24,800 spiking Izhikevich neurons [2], consisting of regular-firing and bursting pyramidal neurons and fast-spiking and low-threshold-spiking interneurons, with connectivities and proportions of each cell type across each cortical layer drawn from empirical mammalian M1 literature. Opsin channel properties were based on empirical estimates. The network model was calibrated to reproduce experimentally observed firing rates and dynamics.

Experimentally, optogenetic stimulation was found to increase firing rates by up to 300 spikes/sec, with higher firing rates observed close to the optrode; firing returned to baseline values between 4 and 5 mm laterally from the optrode. A similar response was observed in the model. Manipulating the strength of synaptic connectivity in the model elucidated several noteworthy aspects of the response. First, the decrease in peak firing rates very near the optrode appears to be due to increased recruitment of inhibitory neurons. Second, even though all excitatory neurons in the model had identical biophysical properties and received identical current input from the opsin, heterogeneities in connectivity were sufficient to account for the broad range of observed firing rates. Third, all of the high-firing excitatory cells (>100 spikes/sec) were opsin-expressing; no indirectly activated excitatory cells fired above 60 spikes/sec.

Applying spectral Granger causality to the LFPs produced by the different cortical layers of the model showed that the strongest projection without stimulation was from layer 2/3 to layer 5A, consistent with the hypothesis that descending excitation is the primary driver of dynamics in the motor cortex. Strong Granger causality from layer 5A to layer 5B was also observed. Across all layer pairs, Granger causality showed a pronounced peak in the mu rhythm band (~9 Hz), with a small, broad bump in the gamma band (~40 Hz) observed in pathways from layer 2/3 to other layers. Optogenetic stimulation in the model increased Granger causality from layer 5 to other layers in a narrow band near the stimulation frequency. It also increased the amplitude of Granger causality from layer 2/3 to other layers in the mu rhythm band, while decreasing it in the gamma band.

In summary, this work demonstrates that (1) synaptic connections play an important role in determining the network-level response to optogenetic stimulation, and (2) optogenetic stimulation may be used to enhance and suppress information flow in particular frequency bands and between particular cortical layers.
